# Framework Membranes for Aqueous Batteries: From Microporous, Mesoporous to Macroporous Structures

**DOI:** 10.1021/acscentsci.5c00365

**Published:** 2025-04-17

**Authors:** Lin Liu, Jiahao Chen, Ahmed Elzatahry, Dongliang Chao, Dongyuan Zhao

**Affiliations:** † Laboratory of Advanced Materials, Aqueous Battery Center, Shanghai Key Laboratory of Molecular Catalysis and Innovative Materials, State Key Laboratory of Molecular Engineering of Polymers, Shanghai Wusong Laboratory of Materials Science, Electron Microscope Center of Fudan University, and Faculty of Chemistry and Materials, 12478 Fudan University, Shanghai, 200433, P. R. China; ‡ William A. Brookshire Department of Chemical and Biomolecular Engineering, 165986 University of Houston, Houston, Texas 77204, United States

## Abstract

To pursue high safety and more affordable energy storage systems, aqueous batteries (ABs) have become a promising contender. Nevertheless, critical challenges persist in diverse AB systems for large-scale applications, including dendrite growth, ion shuttle effects, hydrogen evolution, and corrosion. Notably, owing to the high specific surface area, tunable pore size, and easy multifunctionalization, framework membranes have garnered significant attention as key components for enhancing the performance of ABs. In this context, it is necessary to summarize and discuss the precise pore architecture design of framework membranes in regulating the ionic conductivity and selectivity, to gain an in-depth and comprehensive understanding. The categorization from microporous, mesoporous, to macroporous framework membranes aims to rationally evaluate the structure-performance relationships. Finally, perspectives on the potential applications of framework membranes in ABs are discussed, offering valuable insights for future research and development.

## Graphical abstract

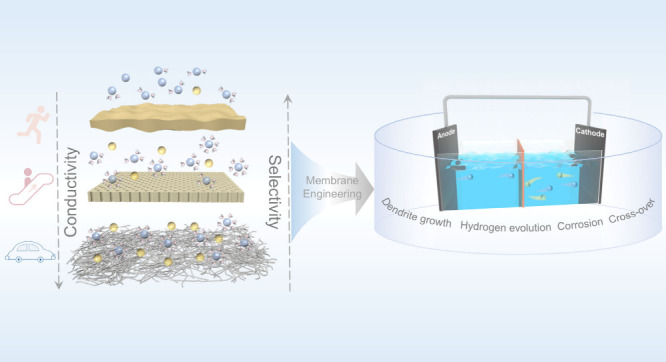

With the access of renewable energy sources to the power grid, there is a growing demand for energy storage systems, which are applied to regulate electricity in peaks and valleys and stabilize the frequency.^1^ Compared to lithium-ion batteries with more flammable organic electrolytes, aqueous batteries (ABs) offer a safe, cost-effective alternative, making them ideal for large-scale energy storage and grid stability.^2,3^ In general, ABs utilize water as a solvent to dissolve soluble salts, thereby forming an ionic-conductive medium. On the anode side, materials such as metallic anodes, carbon-based compounds, or alloy structures are typically employed. Meanwhile, the cathode components commonly incorporate MnO_2_, V_2_O_5_, or organic materials. Nevertheless, ABs are primarily confined by thorny issues, such as narrow window voltage, side reactions at electrodes, uncontrollable dendrite growth, and ion shuttles (Figure 1a).^4,5^ Researchers have explored various approaches, including aqueous hybrid ion batteries, monometallic ion batteries, and aqueous redox flow batteries (ARFBs), to address these challenges. Significant efforts have been dedicated to optimizing anode and cathode materials, electrolytes, separators, and energy storage mechanisms.^6–8^ Practical innovations in any of the above components will promote the tremendous growth of ABs.

**Figure 1:**
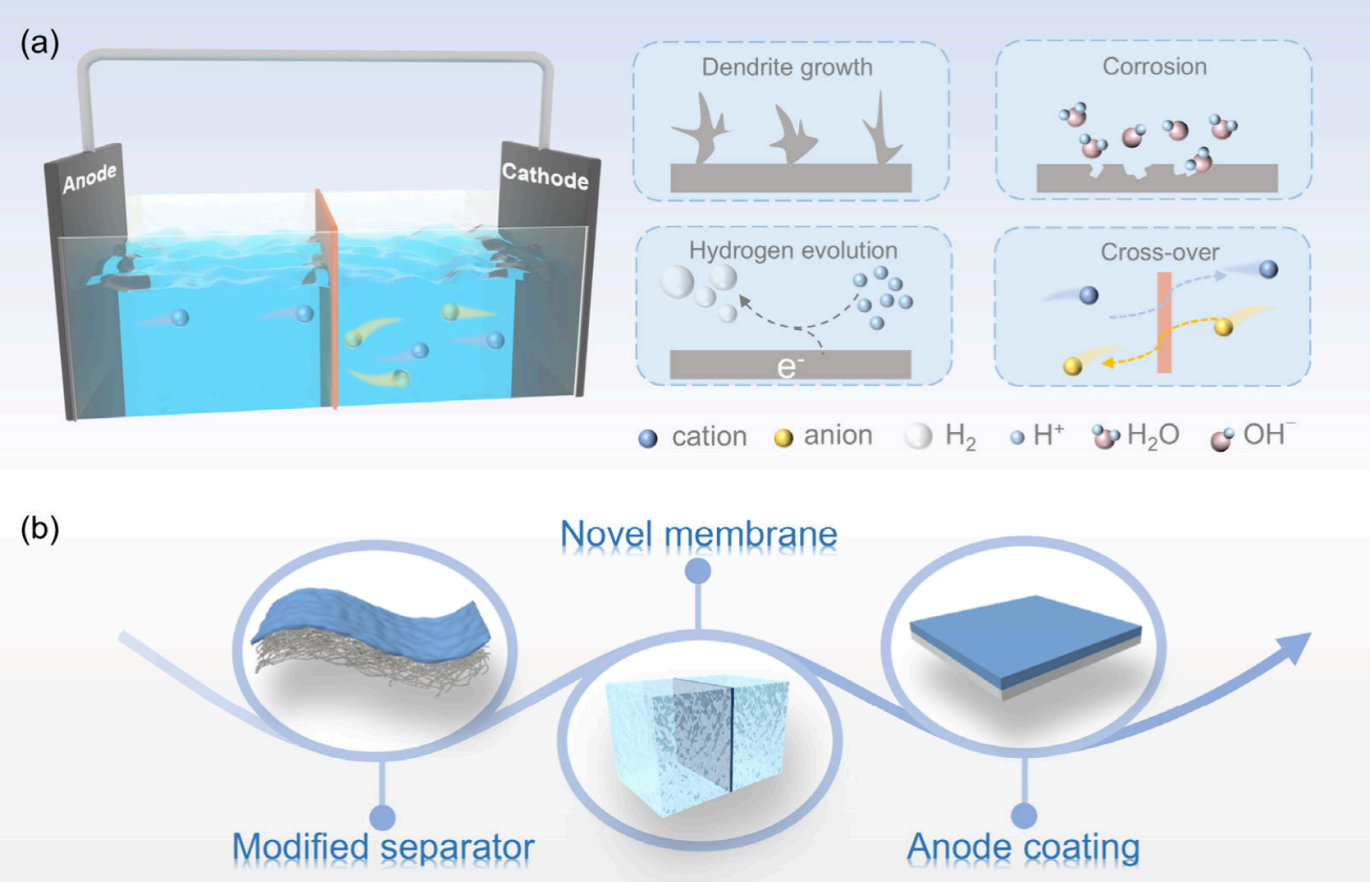
(a) Device composition and critical challenges toward advanced ABs. (b) Major forms of framework membranes in ABs.

Membrane engineering technology plays a pivotal role in battery devices, encompassing components such as separators, ion exchange membranes, and functional coatings. Extensive research efforts have focused on modifying and innovating the membranes in a wide range of ways (Figure 1b).^9–12^ Among them, the main role of the separators is to effectively prevent direct contact between the anode and cathode, thereby avoiding short circuits within the battery. Additionally, they can store electrolytes to maintain stable charging and discharging processes, and form ion-fast channels to ensure uniform ion distribution, effectively inhibiting dendrite growth. Ion exchange membranes are primarily used in ARFBs, which can prevent the active substances from crossing over and promote the rapid migration of specific ions. This functionality is essential for maintaining charge transfer efficiency and ensuring the smooth progression of electrochemical reactions within the battery. Functional coatings usually have multiple roles, such as modulating the concentration field, electric field, and surface binding energy to control the ion and molecular migration behavior and the nucleation potential of the reaction interface, respectively, and to guide the uniform nucleation growth of ions to restrain the side reactions. In brief, the membrane exists as a protective barrier in ABs, and its structure and function directly affect the overall performance and reliability of the battery.

Currently, commercial membranes in batteries are mainly glass fiber as well as Nafion membranes. However, glass fiber membranes suffer from nonuniform pore distribution, which hinders uniform ion deposition. And they are more fragile and unable to withstand the dendrite growth stress. While Nafion membranes are high-cost and prone to swelling in aqueous environments due to their linear polymer structure. Furthermore, the microphase-separated structure is not conducive to analyzing the transport mechanism.^13,14^ Thus, developing novel membrane materials is essential to achieve efficient and stable cycling in ABs.

It is worth noting that porous framework materials have the advantages of high specific surface area, diverse building blocks, rigid backbone structure, and continuous channels, which demonstrate outstanding merits in the field of electrochemical energy storage.^15^ In particular, the pore size and distribution directly affect the properties. Based on the pore size, they can be categorized into microporous (≤2 nm), mesoporous (2–50 nm), and macroporous (≥50 nm) materials. Microporous materials exhibit excellent capabilities for efficient molecular sieving and storage due to their unique pore characteristics. Mesoporous materials typically have relatively larger pore sizes, resulting in lower diffusion resistance and the ability to balance adsorption and diffusion processes to some extent. Macroporous materials, distinguished by their large pore diameters, offer outstanding advantages in electrolyte accommodation and rapid mass transfer. Therefore, these features make porous framework membranes promising candidates for enhancing the performance of ABs.

To the best of our knowledge, there have been many reviews on separator modification,^16,17^ but there is no literature focusing on analyzing the differences in performance of membrane materials in ABs from the perspective of pore characteristics. Therefore, this outlook aims to fill that gap by providing a concise overview of contemporary research on framework membranes, categorized into microporous, mesoporous, and macroporous structures. It highlights the relationship between pore size and battery performance by summarizing their characteristics. It also provides insights into the future structural development of membranes, guiding researchers in developing membranes with targeted functionalities.

## Microporous Framework Membranes

Optimization strategies for microporous framework membranes in ABs are mainly reflected in the ion sieving layers. These membranes are capable of selectively allowing specific ions to pass while blocking others based on the size, charge, shape, and other characteristics of the ions (Figure 2a). Beyond the precise screening of ions, they also exhibit certain ionic conductivity. In ABs, these membranes control ion transport by allowing only target ions to migrate between the anode and cathode, ensuring efficient electrochemical reactions. This selective transport mechanism significantly enhances the efficiency and performance of the battery’s charging and discharging processes. Generally, depending on the bonding type, microporous framework membranes in ABs are categorized into molecular sieve membranes, metal–organic framework (MOF) membranes, and porous organic framework membranes.

**Figure 2:**
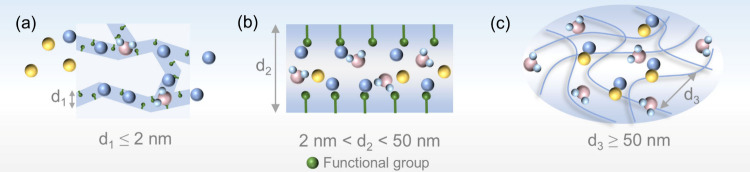
Schematic microstructure of pore action mechanism in ABs: (a) micropore; (b) mesopore; (c) macropore.

The sieving activity of molecular sieve and MOF mainly relies on their pore size. For instance, in the zinc–iodine battery system, the ionic radius of I_3_^–^ (5.14 Å) induces severe shuttle effects, compromising cycling stability. Zhou et al. developed the MOF (Zn-BTC) membrane that combines size-exclusive I_3_^–^ blocking with anode side-reaction suppression. The electrolyte with glass fiber turned dark yellow within 8 h, while Zn-BTC remained colorless even after 7 days. The battery achieves an ultralong 6,000 cycles with 84.6% capacity retention and 99.65% Coulombic efficiency.^18^ Subsequent refinement yielded zeolite membrane with precisely tuned 4 Å pores, delivering 91.0% capacity retention over 30,000 cycles at 4 A g^–1^.^19^ And Fan et al. proposed to construct a solid–liquid mixed electrolyte membrane with molecular sieve (ZSM-5) to breakthrough the structure of separator-free batteries. The microporous channels (5–7 Å) of ZSM-5 with Si–O and Al–O bonds confined the solvated water from free water and zinc ions, which significantly suppressed the side reactions and homogenized the transport of zinc ions. The V_2_O_5_//ZSM-5//Zn full-cell battery can be operated stably for 15,000 cycles (82.4% capacity retention) at 5 A g^–1^.^20^ In contrast, porous organic framework membranes tend to leverage their diverse organic building blocks, prompting them to perform efficient ion selection mostly through charge interactions between functional groups and ions.^21^ Qiao and his colleagues have reported a cost-effective cation exchange membrane (polyPTX) with a rigid polymer backbone.^22^ Its swelling rate is only 5%, much lower than that of Nafion (22.3%). The cost of laboratory-scale preparation of the polyPTX membrane was calculated at US$ 1760 m^–2^, about half the price of Nafion (US$ 3,500 m^–2^). The twisted stacking of the rigid polymer chains endows the membrane microporous framework with the ability to adsorb I_3_^–^ after immersion in concentrated Zn­(I_3_)_2_ solution. Moreover, the – SO_3_H groups within the framework can promote the Zn^2+^ conduction and ensure it flows evenly to the anode interface. The ion conductivity of polyPTX is up to 1.1 mS cm^–1^. It prevents the spontaneous aggregation of Zn^2+^ to the “Tip” resulting in a dendrite-free surface and reducing water-induced corrosion. The battery demonstrates exceptional cycling stability, retaining 96.1% capacity over 10,000 cycles at 2 A g^–1^. In addition, given the solubility of organic framework membranes for large-scale preparation, they are prominent in ion exchange membranes for ARFBs. Recently, Song’s group has successfully developed novel, highly selective ion exchange membranes.^12^ By modifying hydrophobic functional groups to hinder hydration, the mechanism of water and ion conduction in the subnanometer-scale pore-confined environment has been deeply investigated.

Molecular sieve and MOF are predominantly crystalline and show significant advantages in precise size tuning, while organic microporous membranes exhibit excellent structural diversity and membrane-forming capabilities. This enables the microporous framework membranes to have high ion selectivity and strong antiswelling properties. However, excessively small micropore diameters hinder sufficient electrolyte wetting of the separator, restricting ion transport pathways and impeding diffusion. This leads to an increase in the internal resistance of the battery, which affects the charging and discharging efficiency and power performance, posing challenges for achieving optimal energy storage performance.

This enables the microporous framework membranes to have high ion selectivity and strong anti-swelling properties.

## Mesoporous Framework Membranes

Cost-effective mesoporous materials (∼$456 kg^–1^) facilitate rapid ion migration in electrolytes (Figure 2b). Compared to microporous membranes, mesoporous framework membranes exhibit lower resistance to ion transport. Moreover, the mesoporous structure provides moderate specific surface area and pore volume, which not only increases the contact area between the separator and the electrolyte, but also enhances the ion adsorption and transfer efficiency. Furthermore, it can provide more sites for the attachment of electrode materials and enhance the interfacial bonding between the electrode and separator. This strengthened interface facilitates more efficient charge transfer within the battery, ultimately contributing to enhanced overall performance.

Prior studies have demonstrated that mesoporous polyimide membranes can effectively inhibit lithium dendrites.^23^ The mesoporous structure promotes uniform ion deposition, with pore width smaller than the dendrite dimensions, thereby preventing punctures. In the ABs system, the mesoporous membranes can also be used to modify the metal anode. By adjusting the mesopore size (e.g., mesoporous silica, polymers), the solvation structure of the partial electrolyte on the metal surface can be tailored.^24^ Our group has successfully introduced mesoporous membranes into the ABs system.^25^ The Janus mesoporous SiO_2_ interface was assembled on the Zn anode surface with the electrochemical tandem strategy. The rich hydrophilic −OH and hydrophobic −F groups are sequentially constructed in the identical mesopores to decouple and accelerate the Zn^2+^(H_2_O)_6_ cluster trapping and detachment process. Specifically, −OH groups on the surface rapidly captured Zn^2+^ from the bulk-phase electrolyte, while −F groups at the base facilitated the desolvation process. Combined with electrochemical tests, this tandem strategy functions as a “Mesopores Accelerator”, realizing a low energy barrier Zn^2+^ reduction and no side reaction, along with a long lifespan. The symmetric batteries achieved over 8,000 h and 2,000 h of stable plating/stripping at 4 and 10 mA cm^–2^, respectively. Although mesopores are less effective for ion sieving, their regular mesopore channels can confer disordered stacked polymer chains with interconnected networks and more exposed active sites. Researchers have further optimized the type of functional groups within the pores to inhibit the shuttling of actives through strong chemical interactions.^26^ Mesoporous poly­(3,4-ethylenedioxythiophene): poly­(styrenesulfonate) with high electrical conductivity (172.9 S cm^–1^) contains rich sulfur and oxygen functional groups, exhibiting excellent iodine adsorption capacity. Assembled zinc–iodine batteries have excellent multiplication performance and an ultralong cycle life of 20,000 cycles.

Mesoporous framework membranes can reduce transport resistance and accelerate uniform ion deposition through their ordered mesostructure.

Mesoporous framework membranes can reduce transport resistance and accelerate uniform ion deposition through their ordered mesostructure. However, their pore characteristics present certain limitations in practical applications. Although the diffusion of molecules within the pores is relatively smoother, the restriction of ions is relatively inefficient. This leads to the pending considerations in ion exchange membranes for flow-ABs. Moreover, the existence of mesoporous framework membranes is heavily dependent on the substrate. The preparation process is complicated, involving the regulation of the templating agent, solvent, temperature, and other aspects.

## Macroporous Framework Membranes

In addition to superior ionic sieving and conductivity, a high aqueous electrolyte capacity is essential for high-performance separators in ABs. It is obvious that macroporous framework membranes exhibit outstanding advantages in liquid storage compared to microporous and mesoporous membranes (Figure 2c). Recent studies have shown that the integration of two-dimensional (2-D) materials into 3-D macroscopic structures can effectively solve the problem of poor ion and electron transport in electrode materials.^27^ Consequently, great efforts have been made to develop and modify innovative macroporous framework membranes.

Conventional commercial separators are unevenly scaled glass fiber (pore size of ∼280 nm, $680 m^–2^) or nonwoven PP separator (pore size of <5 μm, $55 m^–2^). Modification of separators is one of the simplest and most efficient ways to improve performance. Strategies include the surface modification of mesoporous graphene, silica nanoparticles, and molecular sieves, aiming to homogenize the pores.^10,28,29^ New materials are also an effective means. Pan et al. constructed polyacrylonitrile/graphene oxide (PG) separators with an average diameter of ∼150 nm by electrospinning.^30^ The PG separator exhibits excellent mechanical strength, electrolyte uptake, and hydrophilicity. Notably, it could modulate the solvated structure and migration of Zn^2+^, achieve uniform planar deposition of zinc, and inhibit vertical dendritic growth. The ionic conductivity of the PG separator for Zn^2+^ is as high as 7.69 mS cm^–1^. The battery with PG separator maintains 71.5% of its original capacity after 2,800 cycles at 2 A g^–1^. The tunable building blocks and ordered structure of covalent organic framework (COFs) make them advanced separator candidates. Wang’s group designed a separator using COF with quasi-single-ion conduction.^31^ The COF-Zn membrane has a high zinc ion transference number (0.87), and the thickness of the membrane is only 25 μm. The Zn//4,5,9,10-pyrenetetrone (PTO) cell assembled using COF-Zn can be stable for all-weather cycling in the range of – 40 to +100 °C. In another notable example, Mai and his co-workers introduced macroporous framework membranes to three-dimensional microelectrodes.^32^ They electrodeposited PEDOT-MnO_2_ on 3-D macroporous Ni-framework interdigital microelectrodes. The macroporous structure provides wide-open channels for efficient ion transport and realizes synergy between high energy density and high power density.

Macroporous framework membranes play an important role in ABs to improve electrolyte permeation and ion diffusion performance.

Macroporous framework membranes play an important role in ABs to improve electrolyte permeation and ion diffusion performance. While macropores can provide some benefits, they also present certain limitations. The presence of macropores can reduce the overall structural stability of the membrane, rendering it more vulnerable to mechanical stress or chemical degradation over long-term operation. These factors can compromise both the performance and operational lifetime of the batteries.

To conclude, framework membranes have emerged as pivotal components in the design of advanced ABs. These membranes span a range of pore sizes, including microporous, mesoporous, and macroporous structures, each contributing distinct functionalities to optimize electrochemical performance. Among them, macroporous framework membranes are widely used in separators. New ion exchange membranes are mainly microporous framework membranes. Both mesoporous and microporous framework membranes can be used as coatings to improve battery performance. A focused analysis of pore functionality, as illustrated in Figure 3, can significantly enhance the performance: Micropores for efficient ion sieving, mesopores for fast ion transport, and macropores for electrolyte storage. Accurate regulation of the membrane enables uniform ion deposition and inhibits side reactions and ion shuttling. However, single-component membranes have their own limitations. It highlights the need for the development of innovative and multifunctional membrane materials in future research and development efforts.

1) A comprehensive investigation into the mechanism of structural changes within pore formations is critical. Framework size directly affects ion sieving. Framework charges deeply influence ion transport selectivity through electrostatic interactions, desolvation modulation, and ion exchange kinetics.
2) With the design of multilayer structural membranes, the structure and pore size of different layers can complement each other to improve the overall mechanical and chemical stability. For example, a macroporous framework membrane coated with flexible microporous membrane on the surface can effectively reduce mechanical stress.
3) There is a need to create membrane materials that maintain high performance under extreme conditions (strong acidic or alkaline environments) to ensure versatility across various operational scenarios. For example, all-carbon-linked backbones can be designed to improve stability.
4) Exploring self-supporting membrane design schemes: Introducing polymer chains into channels to improve membrane formation; Alternative gas–liquid interfacial polymerization methods to eliminate substrate dependence.
5) Reducing membrane thickness can effectively shorten ion diffusion paths and improve performance. However, maintaining sufficient mechanical stability is critical to ensure the membrane’s long-term durability under operational stress.
6) Controlling the production costs of advanced membrane technologies is essential for enabling large-scale manufacturing and facilitating their practical application in commercial AB systems.
7) AI-driven membrane screening: Reinforcement learning optimizes polymerization parameters (temperature, solvent ratios) to accelerate synthesis and minimize defects; Machine learning predicts battery performance through chemical/physical property analysis, slashing material screening time/cost.

**Figure 3:**
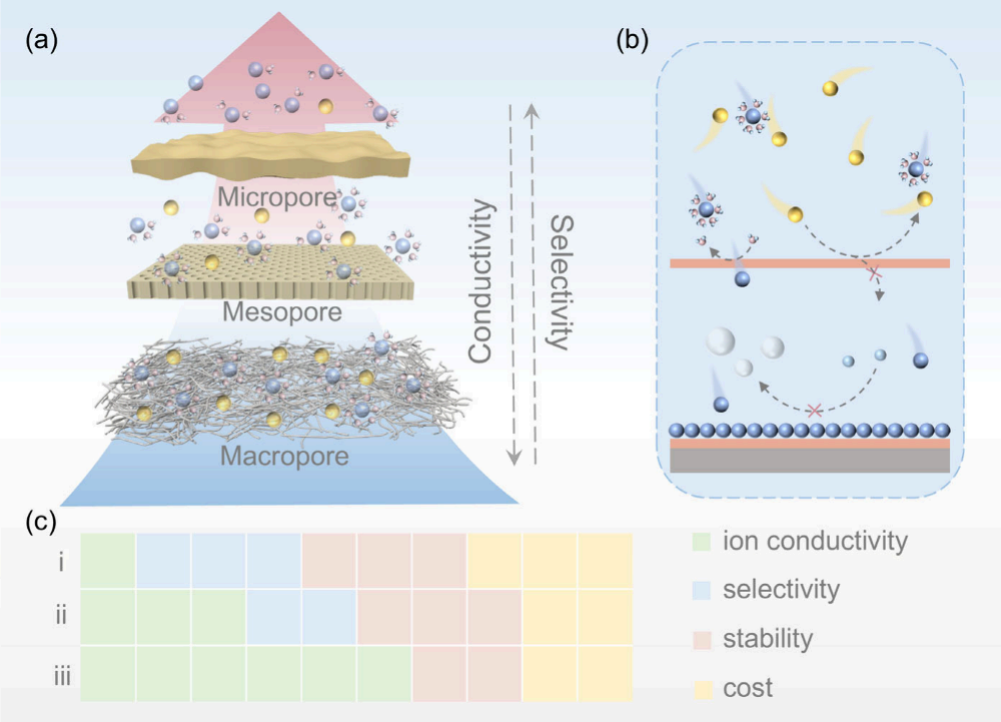
(a) Schematic comparison of ionic conductivity and selectivity of various framework membranes. (b) Schematic diagram of membrane optimization mechanism in ABs. (c) Comparison of the strengths and limitations of framework membrane (i: microporous framework membrane; ii: mesoporous framework membrane; iii: macroporous framework membrane).
